# Dissecting the role of platelet-derived transforming growth factor-β1 in pulmonary fibrosis

**DOI:** 10.1073/pnas.2405287121

**Published:** 2024-10-18

**Authors:** Deborah L. W. Chong, Chris J. Scotton, Joanna C. Porter

**Affiliations:** ^a^Institute for Infection and Immunity, City St George's University of London, London SW17 0RE, United Kingdom; ^b^Department of Respiratory Medicine, University College London, London WC1E 6JF, United Kingdom; ^c^Department of Clinical and Biomedical Science, University of Exeter, Exeter EX1 2LU, United Kingdom

Cellular sources of transforming growth factor-β1 (TGFβ1), a key profibrotic mediator, in pulmonary fibrosis (PF) remain unclear, although alveolar macrophages and fibroblasts have been suggested. Platelets are an abundant source of TGFβ1 and platelet-derived TGFβ1 contributes to wound healing (1). Patients with PF have increased platelet reactivity (2) and depletion of platelets attenuates fibrosis in animal PF models (3). Therefore platelets could represent a source of TGFβ1 in PF. Riehl et al. (4) demonstrated that activated platelets release TGFβ1 to drive lung fibrosis. We have read this article with interest, as some of their findings significantly differ from our study (5).

The authors first showed that patients with idiopathic PF or bleomycin-treated wild-type mice express more citrullinated histone H3 (citH3) than controls. This observation aligns with studies citing the increased presence of citH3-containing neutrophil extracellular traps (NETs) in fibrotic lungs (6). The authors then demonstrated that externalized histones activate platelets to secrete TGFβ. This observation is important, given that previous reports have focused on the direct profibrotic effects of NETs, rather than the cellular networks occurring between NETs and platelets.

The authors then showed that PF was attenuated in bleomycin-treated platelet-specific TGFβ1-deficient mice compared to controls. This finding contrasts with our study, where different PF4-Cre.TGFβ1^fl/fl^ transgenic mice treated with the same bleomycin dose for 28 d had equivalent PF severity as controls (5). We found 0.32% difference in PF between PF4-Cre.TGFβ1^fl/fl^ and littermates by micro-computed tomography (micro-CT) analysis. Therefore, platelet-derived TGFβ1 has a modest biological effect in our model, with an effect size of 0.07. Additionally, although the transgenics used in both studies are assumed to be functionally equivalent, different TGFβ1^fl/fl^ strains were used, which may have influenced the disease outcomes. Furthermore, Riehl et al. used only male mice, while we used both sexes. Sex-specific differences in PF susceptibility are reported in patients and animal models (7). However, reanalyzing our data separately by sex did not reveal any statistically significant impact of platelet-TGFβ1 depletion on disease severity.

Bleomycin remains the gold standard agent to induce PF in animals, although the administration route should be carefully reviewed (8). Riehl et al. administrated bleomycin intratracheally, while we administered bleomycin via an oropharyngeal route. Intratracheal instillation induces a bronchocentric PF pattern, whereas oropharyngeal instillation leads to more homogenous PF distribution over the lung (9). Riehl et al. used digital quantification of tissue sections to assess PF severity. This approach may suffer from sampling bias, especially if the PF distribution is not homogenous throughout the lung. As an alternative, we used micro-CT scanning of the whole lung to provide a more robust quantification of PF severity.

Overall, Riehl et al. postulated a novel interaction between externalized histones and platelets, leading to TGFβ1/interleukin (IL)-27 imbalance to promote PF. Our study directly challenges part of this hypothesis. Despite rigorous attempts, we were unable to demonstrate a major role for platelet-derived TGFβ1 in PF, although IL-27 expression was not interrogated in our model. These contrasting findings highlight the many caveats when using animal PF models, which should all be considered when interpreting such data.
